# Studies on the Chemical Diversities of Secondary Metabolites Produced by *Neosartorya fischeri* via the OSMAC Method

**DOI:** 10.3390/molecules23112772

**Published:** 2018-10-25

**Authors:** You-Min Ying, Lu Huang, Ting Tian, Cui-Yu Li, Shi-Lei Wang, Lie-Feng Ma, Wei-Guang Shan, Jian-Wei Wang, Zha-Jun Zhan

**Affiliations:** 1 College of Pharmaceutical Science, Zhejiang University of Technology, Hangzhou 310014, China; ymying@zjut.edu.cn (Y.-M.Y.); 15958025120@163.com (L.H.); m17816037931@163.com (T.T.); aalicuiyu@163.com (C.-Y.L.); maliefeng@zjut.edu.cn (L.-F.M.); tianranyaowu@zjut.edu.cn (W.-G.S.); 2 College of Biology and Environment Engineering, Zhejiang Shuren University, Hangzhou 310015, China; wangshilei1105@163.com

**Keywords:** OSMAC, pyripyropenes, *Neosartorya fischeri*, secondary metabolites

## Abstract

The One Strain Many Compounds (OSMAC) method was applied to explore the chemical diversities of secondary metabolites produced by *Neosartorya fischeri* NRRL 181. Four pyripyropenes **1**–**4**, eight steroids **5**–**11**, and four prenylated indole alkaloids **12**–**15**, were obtained from the fungus cultured in petri dishes containing potato dextrose agar (PDA). 1,7,11-trideacetylpyripyropene A (**1**) and 1,11-dideacetyl pyripyropene A (**2**) were obtained and spectroscopically characterized (1D, 2D NMR, and HR-ESI-MS) from a natural source for the first time. It offered a sustainable source of these two compounds, which were usually used as starting materials in preparing pyripyropene derivatives. In addition, as compared with all the other naturally occurring pyripyropenes, **1** and **2** possessed unique acetylation patterns that did not follow the established late-step biosynthetic rules of pyripyropenes. The natural occurrence of **1** and **2** in the fungus implied that the timing and order of hydroxylation and acetylation in the late-step biosynthetic pathway of pyripyropenes remained to be revealed. The isolation and identification of **1**–**15** indicated that the OSMAC method could remarkably alter the metabolic profile and enrich the chemical diversities of fungal metabolites. Compounds **1**–**4** exhibited no obvious cytotoxicity against the triple-negative breast cancer cell line MDA-MB-231 as compared with taxol.

## 1. Introduction

Filamentous fungi are prolific producers of bioactive natural products [1], as exemplified by the antibiotic penicillin [2] and the anti-hypercholesterolemia drug lovastatin [3]. In recent years, data from genome sequencing have revealed an inconsistency between the number of biosynthetic gene clusters (BGCs) identified as potentially encoding secondary metabolites and the actual number of chemically characterized secondary metabolites from any given fungi [4]. The reason underlying the phenomenon is that many BGCs are not expressed under laboratory conditions and are generally considered as silent or cryptic. Inducing the expression of these silent BGCs could unlock the chemical diversity they control, allowing the discovery of novel molecules of both medical and biotechnological interest. Therefore, both genetic and cultivation-based techniques have been developed aimed at stimulating expression of these silent BGCs [4]. The principles behind the cultivation based approaches have been conceptualized in the “one strain many compounds” (OSMAC) framework, which underlines how a single strain can produce different molecules when grown under different environmental conditions [5]. Unlike genetic manipulations, the OSMAC approach is not targeted to the activation of a specific cryptic gene cluster, but to the systematic alteration of readily accessible culturing parameters including medium components (salts, amino acids, and carbon source), pH, culture aeration (including the type of culture vessel used), and temperature of growth [6]. This makes the OSMAC approach an accessible, versatile, inexpensive, and relatively simple tool for regulating the metabolism of fungi [6]. 

*Neosartorya fischeri* is a thermotolerance fungus belonging to the *Aspergillus* subgenus *Fumigati* subgroup *Fumigati*, and shares several common physical traits with *Aspergillus fumigatus* [7]. As compared with *A. fumigatus*, the secondary metabolites of *N. fischeri* have not been extensively studied and only resulted in the discovery of limited types of compounds [8]. Nevertheless, bioinformatics prediction by antiSMASH [9] revealed that the genome of *N. fischeri* NRRL181 harbored 39 BGCs, with 25 of them not related to any previously reported natural products (https://fungismash.secondarymetabolites.org/upload/fungi-d6eb54a9-0d96-4fa0-bf97ba8be5065608/index.html), indicating great potential for mining the fungus for novel secondary metabolites. In our previous endeavor at discovering novel structures from fungi, we focused on two *N. fischeri* strains, i.e., *N. fischeri* CGMCC 3.5378 and *N. fischeri* NRRL181. In-depth studies on the secondary metabolites of *N. fischeri* CGMCC 3.5378 led to the isolation of five new prenylated indole alkaloids cultured on wheat bran [10,11] and moist corn [12], while the preliminary studies on *N. fischeri* NRRL181 furnished two new fumiquinazolines and a known compound pyripyropene A [13]. The promising bioactivities and rare natural occurrence of pyripyropenes [14,15] encouraged us to explore the chemical diversities of this kind of natural products. As guided by the OSMAC strategy, *N. fischeri* NRRL181 was cultured in 20 different conditions (Table S1) and analyzed for the production of pyripyropenes by HPLC. Herein, we report the isolation, structure characterization, and bioassay of the induced secondary metabolites from the fungus cultured in petri dishes containing PDA agar.

## 2. Results

**Figure 1:**
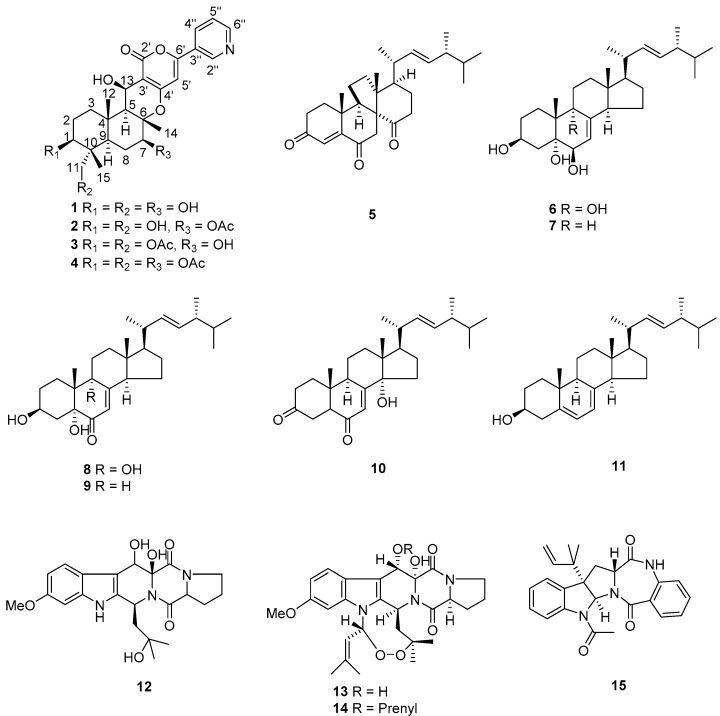
Structures of compounds **1**–**15**.

When *N. fischeri* NRRL181 was cultured on PDA agar in Petri dishes, it gave the most abundant HPLC traces, including several peaks showing the characteristic UV absorption patterns of pyripyropenes (Figure S1). Subsequent chemical investigation on the extract led to the isolation and structure characterization of four pyripyropenes **1**–**4**, eight steroids **5**–**11**, and four prenylated indole alkaloids **12**–**15**. The known compounds **3**–**15** were identified as 7-deacetylpyripyropene A (**3**) [16], pyripyropene A (**4**) [17], dankasterone A (**5**) [18], 22*E*,24*R*-ergosta-7,22-diene-3*β*,5*α*,6*β*,9*α*-tetraol (**6**) [19], 22*E*,24*R*-ergosta-7,22-diene-3*β*,5*α*,6*β*-triol (**7**) [20], 3*β*,5*α*,9*α*-trihydroxy-(22*E*,24*R*)-ergosta-7, 22-dien-6-one (**8**) [21], 3*β*,5*α*-dihydroxy-(22*E*,24*R*)-ergosta-7,22-dien-6-one (**9**) [22,23], (14*α*,22*E*)-14-hydroxyergosta-7,22-diene-3,6-dione (**10**) [24], ergosterol (**11**) [25], 12*β*-hydroxyverruculogen TR-2 (**12**) [26], verruculogen (**13**) [27], fumitremorgin A (**14**) [28], and acetylaszonalenin (**15**) [29] (Figure 1).

**Figure 2:**
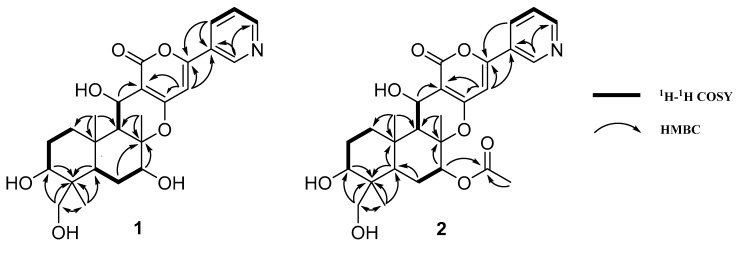
Key ^1^H-^1^H COSY and HMBC correlations in **1** and **2**.

Compound **1** was obtained as a white amorphous powder. The molecular formula was established as C_25_H_31_NO_7_ based on the [M + H]^+^ peak at *m*/*z* 458.2164 given by HR-ESI-MS, corresponding to eleven degrees of unsaturation. UV spectrum showed absorptions at 230 and 319 nm. The IR spectrum exhibited absorption bands for OH (3294 cm^−1^) and carbonyl (1692 cm^−1^) groups. In the ^1^H NMR spectrum of **1**, signals for five olefinic methines at *δ*_H_ 6.86 (s), 7.62 (dd, 8.0, 5.0), 8.32 (ddd, 8.5, 2.0, 1.5), 8.69 (d, 3.5) and 9.07 (s) were readily discerned, along with three O-bearing methines at *δ*_H_ 3.71 (dd, 12.0, 5.0), 3.83 (dd, 11.5, 5.0) and 5.01 (d, 4.0), one O-bearing methylene at *δ*_H_ 3.35 (d, 11.0) and 3.59 (d, 11.0), three methyls at *δ*_H_ 0.79 (s), 1.45 (s), and 1.69 (s). The ^13^C NMR and DEPT spectra of **1** showed 25 resonances attributable to eight quaternary carbons (one carbonyl at *δ*c 165.4, four olefinic at *δ*c 104.4, 129.3, 158.1 and 164.7, one O-bearing at *δ*c 87.0, 10 methines (five olefinic at *δ*c 101.2, 125.5, 134.9, 147.4 and 151.9, and three O-bearing at *δ*c 66.5, 72.9, and 78.4), four methylenes (one O-bearing at *δ*c 66.5) and three methyls. The NMR data of **1** were almost superimposable on those of pyripyropene A (**4**), except for the disappearance of signals ascribable to the three acetyl groups in **4**. These observations, along with the molecular formula, postulated that **1** should be a tri-deacetylated derivative of **4**. A comparison of the chemical shifts and coupling constants of H-1, H-13, and H_2_-11 suggested the deacetyl site at C-1, C-13, and C-11, which was confirmed by comprehensive elucidation of the 2D NMR data (^1^H-^1^H COSY, HSQC, HMBC, and NOESY) (Figure 2) of **1**. Thus, **1** was finally established as 1,7,11-trideacetylpyripyropene A. 

Compound **2** was obtained as a white amorphous powder with a molecular formula of C_27_H_33_NO_8_ according to the [M + H]^+^ peak at *m*/*z* 500.2206 in the HR-ESI-MS mass spectrum. The IR spectrum of **2** also displayed the absorption bands for OH (3335 cm^−1^) and carbonyl (1696 cm^−1^) groups, and the UV spectrum showed absorptions at 230 and 319 nm. In the ^1^H NMR spectrum of **2**, signals ascribable to four methyls at *δ*_H_ 0.79 (s), 1.45 (s), 1.79 (s), and 2.19 (s), one O-bearing methylene at *δ*_H_ 3.31 (d, 11.0) and 3.56 (d, 11.0), three O-bearing methines at *δ*_H_ 3.73 (dd, 12.0, 5.0), 5.08 (dd, 11.5, 5.0), and 5.02 (d, 3.5) can be well distinguished. Similar to **1**, five distinct signals for olefinic CH atoms at *δ*_H_ 6.84 (s), 7.60 (dd, 8.0, 5.0), 8.33 (ddd, 8.0, 2.0, 1.5), 8.67 (dd, 5.0, 2.0), and 9.08 (s) were easily discerned. The ^13^C NMR and DEPT 135 spectrum of **2** showed the presence of four methyls, four methylenes (with one O-bearing at *δ*_C_ 66.7), 10 methines (with three O-bearing at *δ*_C_ 60.3, 72.9, and 80.1, five olefinic at *δ*_C_ 101.1, 125.4, 134.9, 147.4, and 151.9), 10 quaternary carbons (with two carbonyls at *δ*_C_ 172.2 and 165.2, four olefinic at *δ*_C_ 104.5, 129.2, 158.2, and 164.2, and one O-bearing at *δ*_C_ 84.9). The above data exhibited resemblances with **1** and pyripyropene A (**4**), suggesting a pyripyropene skeleton of **2**. As compared to **1**, additional signals (one methyl at *δ*_H_ 2.19 (s) and one carbonyl at *δ*_C_ 172.2) ascribable to an acetyl group were observed. The additional acetyl group was deduced to be harbored at C-7 by comparing the chemical shifts of H-7 and C-7 with those in **1** and **4**, and it was also confirmed by the HMBC correlation from H-7 (*δ*_H_ 5.08 (dd, 11.5, 5.0)) to the carbonyl at *δ*_C_ 172.2. Compound **2** was finally identified as 1,11-dideacetylpyripyropene A on the basis of comprehensive elucidation of the 2D-NMR data (Figure 2). 

As part of our ongoing screening of natural products against triple-negative breast cancer, compounds **1**–**4** were subjected to cytotoxicity assay against MDA-MB-231 cell line by the method we reported previously [30,31]. Unfortunately, none of them showed obvious activity as compared with the positive control, taxol.

## 3. Discussion

In the present work, OSMAC method was used to study the metabolic potential of the fungus *N. fischeri* NRRL181 and the chemical diversity of its secondary metabolites. Along with the targeted isolation of four pyripyropenes **1**–**4**, eight steroids **5**–**11** and four prenylated indole alkaloids **12**–**15** were also identified from the fungus cultured on PDA agar in petri dishes. Compounds **1**–**3** and **5**–**10** have never been obtained in our previous chemical investigations on this fungus and were regarded as the induced metabolites. Compounds **5**–**10** are oxygenated steroids biogenetically related to ergosterol (**11**), with compound **5** featuring a rearranged 6/6/5/6 ring system. Their occurrence herein indicated that silent genes encoding the enzymes for rearrangement and oxidation may be activated in the present culturing condition and lead to the production of this series of oxygenated steroids.

Pyripyropenes are meroterpenoids first isolated from *Aspergillus fumigatus* by the Ōmura group [17]. To date, only 25 pyripyropenes bearing different substitution (hydroxyl, acetoxyl, or propionyloxy groups) at C-1, C-7, C-11 and C-13 of the terpenoid moiety have been obtained from natural sources, with pyripyropenes A–R from *A. fumigatus* FO-1289 and its mutant [17,32,33], pyripyropenes S–T from *A. similanensis* [34,35], 1-deacetylpyripyropene A, 11-deacetylpyripyropene O and 13-dehydroxy-1,11-deacetylpyripyropene A from *Fusarium lateritium* [36], 7-deacetylpyripyropene A (**3**) and 13-dehydroxylpyripyropene A from *N. pseudofischeri* [16]. Pyripyropenes, in particular pyripyropene A (**4**), were discovered as a highly potent selective inhibitor of the ACAT-2 isoform, which was considered a new therapeutic target for the treatment and prevention of hypercholesterolemia and atherosclerosis [14]. The in vivo efficacy of pyripyropene A (**4**) has also been proved [37]. Recently, pyripyropene A (**4**) has also gained attention as a promising lead for developing insecticides since it exhibited excellent insecticidal activity against aphids by both foliar application and soil drenching without serious toxicological issues [15,38,39]. Compounds **1** and **2** were previously prepared by chemical deacetylation of pyripyropene A (**4**) [40,41] and used as starting materials in preparing pyripyropene derivatives. This is the first report on the isolation of the two compounds from a natural source, and the spectroscopic data were also completely reported and precisely assigned for the first time. The acquisition of them enriched the limited structure diversity of naturally occurring pyripyropenes and offered a sustainable way of preparing the two compounds. The BGC of pyripyropenes (*pyr* cluster) was identified in *A. fumigatus* Af293 by Itoh et al. [42], containing nine genes encoding one CoA ligase (*pyr1*), one polyketide synthase (*pyr2*), one terpene cyclase (*pyr4*), one flavin adenine dinucleotide dependent monooxygenase (*pyr5*), one prenyltransferase (*pyr6*), two cytochrome P450s (*pyr3* and *pyr9*), and two acetyltransferases (*pyr7* and *pyr8*). They also precisely proved the early steps in pyripyropenes biosynthesis, in which *pyr1*, *pyr2*, *pyr6*, *pyr5*, and *pyr4* worked sequentially to construct the meroterpenoid core of pyripyropenes (deacetyl-pyripyropene E). However, the hydroxylation and acetylation mechanisms for the late steps were left unresolved. Hu et al. [43,44] identified the second BGC of pyripyropenes (*ppb* cluster) in another pyripyropene A producing strain *Penicillium coprobium* PF1169, where four tailoring genes *ppb3*, *ppb4*, *ppb8*, and *ppb9* were highly homologous to *pyr3*, *pyr9*, *pyr7*, and *pyr8*, respectively. By introducing the four tailoring genes individually into the model fungus *A. oryzae* and feeding the transformants with certain predicted intermediates, they reported the functions of *ppb3* (P450-1), *ppb4* (P450-2), *ppb8* (AT-1), and *ppb9* (AT-2), and proposed the mechanism for the late steps of pyripyropene biosynthesis [43,44]. According to the pathway they proposed, compounds **1** and **2** obtained in the present study were not on the pathway and should be considered as shunt products. However, given that the conclusion has been drawn based on the bioconversion of some predicted intermediates, it is reasonable to infer that one might get different results and depict different pathways if different intermediates were used. The natural occurrence of **1** and **2** in *N. fischeri* NRRL181 offered us with clues that the timing and order of hydroxylation and acetylation in the late-step biosynthetic pathway of pyripyropenes may be different from those reported. Hence, the mechanisms underlying these biosynthetic steps are still open and warrant further in vitro and in vivo studies. 

## 4. Materials and Methods

### 4.1. General Experimental Procedure

All solvents used were of analytical grade and obtained from commercial sources. Solvents were filtered through a microporous membrane of 0.45 μm before used for HPLC analyses. TLC: precoated silica gel GF_254_ plates (Qingdao Marine Chemical Co., Ltd., Qingdao, China); visualized by UV light and spraying with 10% H_2_SO_4_ in 95% EtOH followed by heating. Column chromatography (CC): silica gel (SiO_2_; 200–300 mesh; Qingdao Marine Chemical Co., Ltd.), MCI CHP20P gel (75–150 μm; Mitsubishi Chemical Industries Ltd., Tokyo, Japan) and ODS C-18 gel (50 μm; YMC Co., Ltd., Kyoto, Japan). Optical rotations: Rudolph Research Autopol III automatic polarimeter. UV Spectra: Shimadzu-UV-2450 spectrometer; λ_max_ (log ε) in nm. IR Spectra: Thermo-Nicolet-6700 FT-IR microscope instrument (FT-IR microscope transmission). NMR spectra: Bruker AM-500 apparatus with chemical shifts given in ppm (*δ*) using TMS as an internal standard, J in Hz. ESI- and HR-ESI-MS: Agilent-6210-LC/TOF mass spectrometer; in m/z.

### 4.2. Fungus and Culture Conditions 

The fungus was purchased from DSMZ (DE-Braunschweig). The cultivation was carried out on static potato dextrose agar (PDA) medium at 30 °C for 25 days.

### 4.3. Extraction and Isolation 

The cultivated PDA medium along with the *N. fischeri* NRRL 181 mycelium was successively extracted with methanol (seven days each). The solvent was evaporated under reduced pressure to give a crude extract (13.8 g), which was then subjected to CC (MCI, MeOH/H_2_O 20:80→90:10) to offer 10 fractions Frs. A–J. Fr. D and F were found to contain peaks with characteristic UV absorptions of pyripyropenes and were subjected to separation in priority. Fr. D was purified by CC (ODS C-18, MeOH/H_2_O 45:55→65:35) to give six sub-fractions Frs. D1-D6. Fr. D6 was then separated by CC (ODS C-18; MeOH/H_2_O 60:40) to furnish **3** (10.1 mg). Fr. F was separated by CC (ODS C-18, MeOH/H_2_O 60:40→90:10) into six sub-fractions Frs. F1-F6. Fr. F5 was purified by CC on ODS C-18 (MeOH/H_2_O 65:35→70:30) to give **1** (3.0 mg), **2** (10.0 mg), and **4** (11.1 mg). Fr.F2 was purified by CC (ODS C-18; MeOH/H_2_O 60:40) to give **15** (6.4 mg), and Fr. F6 was separated by CC (silica gel, petroleum ether/acetone 2:1) to furnish **6** (9.1 mg). Fr. B was purified by CC (silica gel, petroleum ether/acetone 3.5:1→1:1) followed by CC (ODS C-18, MeOH/H_2_O 70:30) to furnish **12** (7.8 mg). Fr. G was divided into 10 sub-fractions Fr. G1–G7 by CC (ODS C-18, MeOH/H_2_O 70:30→90:10). **13** (35.4 mg) was crystallized from Fr. G2, while **8** (12.7 mg) and **10** (4.5 mg) was obtained from Fr. G4 by CC (silica gel, petroleum ether/acetone 5:1→3:1). Repeated CC purification (silica gel, petroleum ether/acetone 3:1→2:1) of Fr. G5 furnished **7** (6.7 mg) and **9** (12.3 mg). Fr. H was first separated by CC (ODS C-18, MeOH/H_2_O 75:25→90:10) to give five sub-fractions Fr. H1–H5. **5** (41.3 mg) and **14** (23.6 mg) were crystallized from Fr. H2 and Fr. H4, respectively. **11** (183 mg) was directly crystallized from Fr. J. 

1,7,11-trideacetylpyripyropene A (**1**): white amorphous powder. ${\left[\alpha \right]}_{D}^{25}$ = +40.0 (c = 0.01, MeOH). UV λ_max_ (log *ε*) (MeOH): 320 (0.8), 230 (1.0) 204 (0.8). IR (needle): 3294, 2957, 2924, 2876, 1692, 1579, 1410, 1296, 1045. ^1^H and ^13^C NMR: see Table 1. HR-ESI-MS [M + H]^+^ *m*/*z* 458.2164 (calcd for C_25_ H_32_NO_7_ 458.2173). 

1,11-dideacetyl pyripyropene A (**2**): white amorphous powder. ${\left[\alpha \right]}_{D}^{25}$ = +63.3 (c = 0.01, MeOH). UV λ_max_ (log *ε*) (MeOH): 321 (1.2), 230 (1.4) 203 (1.3). IR (needle): 3335, 2925, 1696, 1584, 1415, 1246, 1037. ^1^H and ^13^C NMR: see Table 1. HR-ESI-MS [M + H]^+^ *m/z* 500.2206 (calcd for C_27_H_34_NO_8_ 500.2217). 

### 4.4. Bioassay

All isolates were evaluated for the cytotoxic activities against MDA-MB-231 cell line according to protocols we previously reported [30,31], employing taxol as the positive control.

## 5. Conclusions

By applying the OSMAC method to *N. fischeri* NRRL 181, four pyripyropenes, eight steroids, and four prenylated indole alkaloids, were obtained from the fungus cultured in petri dishes containing PDA medium. The results validated the effectiveness of the OSMAC method in diversifying the fungal secondary metabolites. The acquisition of the two new naturally-occurring pyripyropene derivatives may help to understand the late-step biosynthetic mechanism of pyripyropenes. 

## Figures and Tables

**Table 1:** ^1^H and ^13^C NMR data of compounds **1** and **2** in CD_3_OD (δ in ppm, *J* in Hz) ^a^.

Position	1		2	
	*δ*_H_	*δ*_C_	*δ*_H_	*δ*_C_
1	3.71 (dd, 12.0, 5.0)	72.9	3.73 (dd, 12.0, 5.0)	72.9
2	1.80 (m)	27.3	1.72 (m)	27.2
	1.90 (m)		1.93 (m)	
3	1.34 (m)	37.7	1.37 (m)	37.5
	2.16 (m)		2.17 (m)	
4	-	39.2	-	39.1
5	1.44 (d, 4.0)	55.7	1.47 (d, 4.0)	55.8
6	-	87.0	-	84.9
7	3.83 (dd, 11.5, 5.0)	78.4	5.08 (dd, 11.5, 5.0)	80.1
8	1.63 (m)	28.9	1.44 (m)	26.5
	1.90 (m)		1.84 (m)	
9	1.51 (dd, 12.5, 1.5)	46.4	1.53 (d, 2.0)	46.3
10	-	43.3	-	43.4
11	3.35 (d, 11.0)	66.5	3.31 (d, 11.0)	66.7
	3.59 (d, 11.0)		3.56 (d, 11.0)	
12	1.45 (s)	18.0	1.45 (s)	17.9
13	5.01 (d, 4.0)	60.5	5.02 (d, 3.5)	60.3
14	1.69 (s)	16.0	1.79 (s)	17.0
15	0.79 (s)	12.7	0.79 (s)	12.6
16	-	-	-	172.2
17	-	-	2.19 (s)	21.2
18	-	-	-	-
19	-	-	-	-
2′	-	165.4	-	165.2
3′	-	104.4	-	104.5
4′	-	164.7	-	164.2
5′	6.86 (s)	101.2	6.84 (s)	101.1
6′	-	158.1	-	158.2
2″	9.07 (brs)	147.4	9.08 (d, 2.0)	147.4
3″	-	129.3	-	129.2
4″	8.32 (ddd, 8.0, 2.0, 1.5)	134.9	8.33 (ddd, 8.0, 2.0, 1.5)	134.9
5″	7.62 (dd, 8.0, 5.0)	125.5	7.60 (dd, 8.0, 5.0)	125.4
6″	8.69 (brd, 3.5)	151.9	8.67 (dd, 5.0, 2.0)	151.9

^a^ 500 MHz for ^1^H and 125 MHz for ^13^C NMR.

## Supplementary Materials

The following are available online at http://www.mdpi.com/1420-3049/23/11/2772/s1. Culturing media screened in the present study (Table S1) and the NMR, HRMS, IR, and UV spectra of compounds **1** and **2** (Figures S1–S16).

## References

[1] Schueffler A., Anke T. (2014). Fungal natural products in research and development. Nat. Prod. Rep..

[2] Ozcengiz G., Demain A.L. (2013). Recent advances in the biosynthesis of penicillins, cephalosporins and clavams and its regulation. Biotechnol. Adv..

[3] Mulder K.C.L., Mulinari F., Franco O.L., Soares M.F., Magalhães B.S., Parachin N.S. (2015). Lovastatin production: From molecular basis to industrial process optimization. Biotechnol. Adv..

[4] Romano S., Jackson S.A., Patry S., Dobson A.D.W. (2018). Extending the “One Strain Many Compounds” (OSMAC) principle to marine microorganisms. Mar. Drugs.

[5] Bode H.B., Bethe B., Höfs R., Zeeck A. (2002). Big effects from small changes: Possible ways to explore nature’s chemical diversity. ChemBioChem..

[6] Zarins-Tutt J.S., Barberi T.T., Gao H., Mearns-Spragg A., Zhang L.X., Newman D.J., Goss R.J.M. (2016). Prospecting for new bacterial metabolites: A glossary of approaches for inducing, activating and upregulating the biosynthesis of bacterial *cryptic* or *silent* natural products. Nat. Prod. Rep..

[7] Girardin H., Monod M., Latgé J.P. (1995). Molecular characterization of the food-borne fungus *Neosartorya fischeri* (Malloch and Cain). Appl. Environ. Microb..

[8] Frisvad J.C., Larsen T.O. (2016). Extrolites of *Aspergillus fumigatus* and other pathogenic species in *Aspergillus* section *Fumigati*. Front. Microbiol..

[9] Weber T., Blin K., Duddela S., Krug D., Kim H.U., Bruccoler R., Lee S.Y., Fischbach M.A., Müller R., Wohlleben W. (2015). antiSMASH 3.0-a comprehensive resource for the genome mining of biosynthetic gene clusters. Nucleic Acids Res..

[10] Zheng Z.Z., Shan W.G., Wang S.L., Ying Y.M., Ma L.F., Zhan Z.J. (2014). Three new prenylated diketopiperazines from Neosartorya fischeri. Helv. Chim. Acta.

[11] Chen B.Y., Wang Z., Ying Y.M., Jiang L.X., Zhan Z.J., Wang J.L., Zhang W., Neofipiperzine D. (2014). A new prenylated indole alkaloid metabolite of the fungus *Neosartorya fischeri*. J. Chem. Res..

[12] Shan W.G., Wang S.L., Ying Y.M., Ma L.F., Zhan Z.J. (2014). Indole-benzodiazepine-2,5-dione derivatives from *Neosartorya fischeri*. J. Chem. Res..

[13] Shan W.G., Wang S.L., Lang H.Y., Chen S.M., Ying Y.M., Zhan Z.J. (2015). Cottoquinazolines E and F from *Neosartorya fischeri* NRRL 181. Helv. Chim. Acta.

[14] Ohshiro T., Rudel L.L., Ōmura S., Tomoda H. (2007). Selectivity of microbial acyl-CoA: Cholesterol acyltransferase inhibitors toward isozymes. J. Antibiot..

[15] Horikoshi R., Goto K., Mitomi M., Oyama K., Sunazuka T., Ōmura S. (2017). Identification of pyripyropene A as a promising insecticidal compound in a microbial metabolite screening. J. Antibiot..

[16] Lan W.J., Fu S.J., Xu M.Y., Liang W.L., Lam C.K., Zhong G.H., Xu J., Yang D.P., Li H.J. (2016). Five new cytotoxic metabolites from the marine fungus *Neosartoya pseudofischri*. Mar. Drugs.

[17] Ōmura S., Tomoda H., Kim Y.K., Nishida H. (1993). Pyripyropenes, highly potent inhibitors of acyl-CoA:cholesterol acyltransferase produced by *Aspergillus fumigatus*. J. Antibiot..

[18] Amagata T., Tanaka M., Yamada T., Doi M., Minoura K., Ohishi H., Yamori T., Numata A. (2007). Variation in cytostatic constituents of a sponge-derived *Gymnascella dankaliensis* by manipulating the carbon source. J. Nat. Prod..

[19] Chen P., Wu J., Dai H.F., Xie X.C., Mei W.L. (2008). Chemical constituents from *Cephalotaxus* endophytic fungus S26 of hainanensis. Chin. J. Med. Chem..

[20] Piccialli V., Sica D. (1987). Four new trihydroxylated sterols from the sponge *Spongionella gracillis*. J. Nat. Prod..

[21] Xiong H.Y., Fei D.Q., Zhou J.S., Yang C.J., Ma G.L. (2009). Steroids and other constituents from the mushroom *Armillaria lueovirens*. Chem. Nat. Compd..

[22] Ishizuka T., Yaoita Y., Kikuchi M. (1997). Sterol constituents from the fruit bodies of *Grifola frondosa* (Fr.) S. F. Gray. Chem. Pharm. Bull..

[23] Yang S.P., Xu J., Yue J.M. (2003). Sterols from the fungus *Catathelasma imperiale*. Chin. J. Chem..

[24] Huang H.C., Liaw C.C., Yang H.L., Hseu Y.C., Kuo H.T., Tsai Y.C., Chien S.C., Amagaya S., Chen Y.C., Kuo Y.H. (2012). Lanostane triterpenoids and sterols from *Antrodia camphorate*. Phytochemistry.

[25] Kang J., Wang H.Q., Chen R.Y. (2003). Studies on the constituents of the mycelia produced from fermented culture of *Flammulina velutipes*. Int. J. Med. Mushrooms.

[26] Li X.J., Zhang Q., Zhang A.L., Gao J.M. (2012). Metabolites from *Aspergillus fumigatus*, an endophytic fungus associated with melia azedarach, and their antifungal, antifeedant and toxic activities. J. Agric. Food Chem..

[27] Afiyatullov S.S., Kalinovskii A.I., Pivkin M.V., Dmitrenok P.S., Kuznetsova T.A. (2004). Fumitremorgins from the marine isolate of the fungus *Aspergillus fumigatus*. Chem. Nat. Compd..

[28] Yamazaki M., Fujimoto H., Kawasaki T. (1980). Chemistry of tremorogenic metabolites. І. Fumitremorgin A from *Aspergillus fumigatus*. Chem. Pharm. Bull..

[29] Kimura Y., Hamasaki T., Nakajima H., Isogai A. (1982). Structure of aszonalenin, a new metabolite of *Aspergillus zonatus*. Tetrahedron Lett..

[30] Tang L., Fu L.L., Lu C.H., Hou X.R., Shan W.G., Zhan Z.J. (2017). New cytotoxic phloroglucinol derivatives from *Agrimonia pilosa*. Fitoterapia.

[31] Zhao H., Wu R., Ma L.F., Wo L.K., Hu Y.Y., Chen C., Zhan Z.J. (2016). Aurovertin-type polyketides from *Calcarrisporium arbuscular* with potent cytotoxic activities against triple-negative breast cancer. Helv. Chim. Acta.

[32] Tomoda H., Tabata N., Yang D.J., Takayanagi H., Nishida H., Ōmura S. (1995). Pyripyropenes, novel ACAT inhibitors produced by *Aspergillus fumigatus* Ⅲ. Structure elucidation of pyripyropenes E. to L.. J. Antibiot..

[33] Tomoda H., Tabata N., Yang D.J., Namatame I., Tanaka H., Ōmura S. (1996). Pyripyropenes, novel ACAT inhibitors produced by *Aspergillus fumigatus* Ⅳ. Structure elucidation of pyripyropenes M. to R. J. Antibiot..

[34] Prompanya C., Dethoup T., Bessa L.J., Pinto M.M.M., Gales L., Costa P.M., Silva A.M.S., Kijjoa A. (2014). New isocoumarin derivatives and meroterpenoids from the marine sponge-associated fungus *Aspergillus similanensis* sp. Nov. KUFA 0013. Mar. Drugs.

[35] Prompanya C., Fernandes C., Cravo S., Pinto M.M.M., Dethoup T., Silva A.M.S., Kijjoa A. (2015). A new cyclic hexapeptide and a new isocoumarin derivative from the marine sponge-associated fungus *Aspergillus similanensis* sp. Nov. KUFA 0013. Mar. Drugs.

[36] Cao Q.X., Wei J.H., Deng R., Feng G.K., Zhu X.F., Lan W.J., Li H.J. (2017). Two new pyripyropenes from the marine fungus Fusarium lateritium 2016F18-1. Chem. Biodivers..

[37] Ohshiro T., Ohtawa M., Nagamitsu T., Matsuda D., Yagyu H., Davis M.A., Rudel L.L., Ishibashi S., Tomaoda H. (2015). New pyripyropene A. derivatives, highly SOAT2-selective inhibitors, improve hypercholesterolemia and atherosclerosis in athrogenic mouse models. J. Pharmacol. Exp. Ther..

[38] Fuse S., Matsumura K., Johmoto K., Uekusa H., Tanaka H., Hirose T., Sunazuka T., Ōmura S., Takahashi T. (2016). The design, synthesis, and evaluation of 1,5,7-trisubstituted-3-pyridyl-xathones for use as insecticides starting from pyripropene A. Chem. Eur. J..

[39] Goto K., Horikoshi R., Mitomi M., Oyama K., Hirose T., Sunazuka T., Ōmura S. (2018). Synthesis and insecticidal efficacy of pyripyropene derivatives focusing on the C-1, C-7, and C-11 positions’ substituent groups. J. Antibiot..

[40] Obata R., Sunazuka T., Li Z.R., Tian Z.M., Harigaya Y., Tabata N., Tomoda H., Ōmura S. (1996). Chemical modification and structure-activity relationship of pyripyropenes 1. Modification at the four hydroxyl groups. J. Antibiot..

[41] Obata R., Sunazuka T., Harigaya Y., Hayashi M., Rho M.C., Tomoda H., Ōmura S. (2000). Structure-activity relationships study of pyripyropenes: Reversal of cancer cell multidrug resistance. J. Antibiot..

[42] Itoh T., Tokunaga K., Matsuda Y., Fujii I., Abe I., Ebizuka Y., Kushiro T. (2010). Reconstitution of a fungal meroterpenoid biosynthesis reveals the involvement of a novel family of terpene cyclase. Nat. Chem..

[43] Hu J., Okawa H., Yamamoto K., Oyama K., Mitomi M., Anzai H. (2011). Characterization of two cytochrome P450 monoxygenase genes of the pyripyropene biosynthetic gene cluster from *Penicillium coprobium*. J. Antibiot..

[44] Hu J., Furutani A., Yamamoto K., Oyama K., Mitomi M., Anzai H. (2014). Characterization of two acetyltransferase genes in the pyripyropene biosynthetic gene cluster from *Penicillium coprobium*. Biotechnol. Biotec. Equip..

