# Supplementary material for: Studies on the Chemical Diversities of Secondary Metabolites Produced by Neosartorya fischeri via the OSMAC Method
Source: Molecules. 2018 Oct 25;23(11):2772. doi: 10.3390/molecules23112772 (PMC6278566; doi:10.3390/molecules23112772)
Supplement: Supplementary file 1 [file molecules-23-02772-s001.pdf]

Studies on the Chemical Diversities of Secondary Metabolites Produced by *Neosartorya fischeri* via the OSMAC Method

You-Min Ying[1], Lu Huang[1], Ting Tian[1], Cui-Yu Li[1], Shi-Lei Wang[2], Lie-Feng Ma[1], Wei-Guang Shan[1], Jian-Wei Wang[*1] and Zha-Jun Zhan[*1]

[1] College of Pharmaceutical Science, Zhejiang University of Technology, 310014, Hangzhou, P. R. China; ymying@zjut.edu.cn (Y. Y.); 1944398951@qq.com (L. H.); 2353703578@qq.com (T. T.); 452896473 @qq.com (C. L.); maliefeng@zjut.edu.cn (L. M.); tianranyaowu@zjut.edu.cn (W. S.); zjnpr@zjut.edu.cn (Z. Z.); wangjianwei@zjut.edu.cn (J. W.)

[2] College of Biology and Environment Engineering, Zhejiang Shuren University, 310015, Hangzhou, P.R. China; wangshilei1105@163.com

* Correspondence: zjnpr@zjut.edu.cn (Z. Z.); wangjianwei@zjut.edu.cn (J. W.) Tel.: +86-571-88871030 (Z. Z.); +86-571-88871075 (J. W.)

Contents

Table S1. Culturing media screened in the present study..........................3

Figure S1. HPLC Traces of the crude extract and purified pyripyropenes.4

Figure S4. ^{1}H-^{1}H COSY Spectrum of 1 in MeOD....................................6

Figure S8. HR-ESI-MS Spectrum of 1...8

Figure S9. IR Spectrum of 1 8

Figure S10. UV Spectrum of 1 in MeOD...9

Figure S11. ^{1}H NMR Spectrum of 2 in MeOD..9

Figure S12. ^{13}C NMR Spectrum of 2 in MeOD.....................................10

Figure S13. ^{1}H-^{1}H COSY Spectrum of 2 in MeOD...............................10

Figure S14. HSQC Spectrum of 2 in MeOD 11

Figure S15. HMBC Spectrum of 2 in MeOD 11

Figure S16. NOESY Spectrum of 2 in MeOD.......................................12

Figure S17. HR-ESI-MS Spectrum of 2...12

Figure S18. IR Spectrum of 2 13

Figure S19. UV Spectrum of 2...13

Table S1. Culturing media screened in the present study.

No.	Media component (in 1 L distilled water)	Liquid /Solid	Vessel type
1#	glycerol 20 g, glucose 5 g, soybean 20 g, yeast extract 2 g, NaCl 2.5 g, $CaCO_3$ 4 g	L	conical flask
2#	glucose 20 g, peptone 20 g, glycine 5 g, K_2HPO_4 2 g, MgSO4 1 g	L	conical flask
3#	glucose 20 g, malt extract 10 g, yeast extract 4 g, agar 20 g	S	Petri dish
4#	$NaNO_3$ 3 g, $MgSO_4 \cdot 7H_2O$ 0.5 g, KCl 0.5 g, $FeSO_4 \cdot 4H_2O$ 0.01 g, K_2HPO_4 1 g, sucrose 30 g, agar 20 g	S	Petri dish
5#	$NaNO_3$ 3.3 g, $MgSO_4 \cdot 7H_2O$ 0.05 g, KCl 0.05 g, $FeSO_4 \cdot 4H_2O$ 0.001 g, K_2HPO_4 1.3 g, yeast extract 5 g, sucrose 30 g, agar 20 g	S	Petri dish
6#	potato 200 g, glucose 20 g, agar 20 g	S	Petri dish
7#	soluble starch 20 g, peptone 10 g, agar 20 g	S	Petri dish
8#	$NaNO_3$ 3 g, $MgSO_4 \cdot 7H_2O$ 0.5 g, KCl 0.5 g, $FeSO_4 \cdot 4H_2O$ 0.01 g, K_2HPO_4 1 g, sucrose 30 g	L	conical flask
9#	$NaNO_3$ 3.3 g, $MgSO_4 \cdot 7H_2O$ 0.05 g, KCl 0.05 g, $FeSO_4 \cdot 4H_2O$ 0.001 g, K_2HPO_4 1.3 g, yeast extract 5 g, sucrose 30 g	L	conical flask
10#	potato 200 g, glucose 20 g	L	conical flask
11#	sucrose 150 g, yeast extract 20 g, $MgSO_4 \cdot 7H_2O$ 0.5 g, $ZnSO_4 \cdot 7H_2O$ 10 mg, trace element ($CuSO_4 \cdot 5H_2O$ 0.4 mg, $Na_2B_4O_7 \cdot 10H_2O$ 0.05 mg, $FeSO_4 \cdot 7H_2O$ 0.8 mg, $MnSO_4 \cdot 2H_2O$ 0.8 mg, $Na_2MoO_4 \cdot 2H_2O$ 0.8 mg, $ZnSO_4 \cdot 7H_2O$ 0.8 mg)	L	conical flask
12#	9# supplemented with 5-aza-2'-deoxycytidine (final concentration: 50 µM)	L	conical flask
13#	9# supplemented with 5-aza-2'-deoxycytidine (final concentration: 100 µM)	L	conical flask
14#	9# supplemented with 5-aza-2'-deoxycytidine (final concentration: 300 µM)	L	conical flask
15#	10# supplemented with 5-aza-2'-deoxycytidine (final concentration: 50 µM)	L	conical flask
16#	10# supplemented with 5-aza-2'-deoxycytidine (final concentration: 100 µM)	L	conical flask
17#	10# supplemented with 5-aza-2'-deoxycytidine (final concentration: 300 µM)	L	conical flask
18#	11# supplemented with 5-aza-2'-deoxycytidine (final concentration: 50 µM)	L	conical flask
19#	11# supplemented with 5-aza-2'-deoxycytidine (final concentration: 100 µM)	L	conical flask
20#	11# supplemented with 5-aza-2'-deoxycytidine (final concentration: 300 µM)	L	conical flask

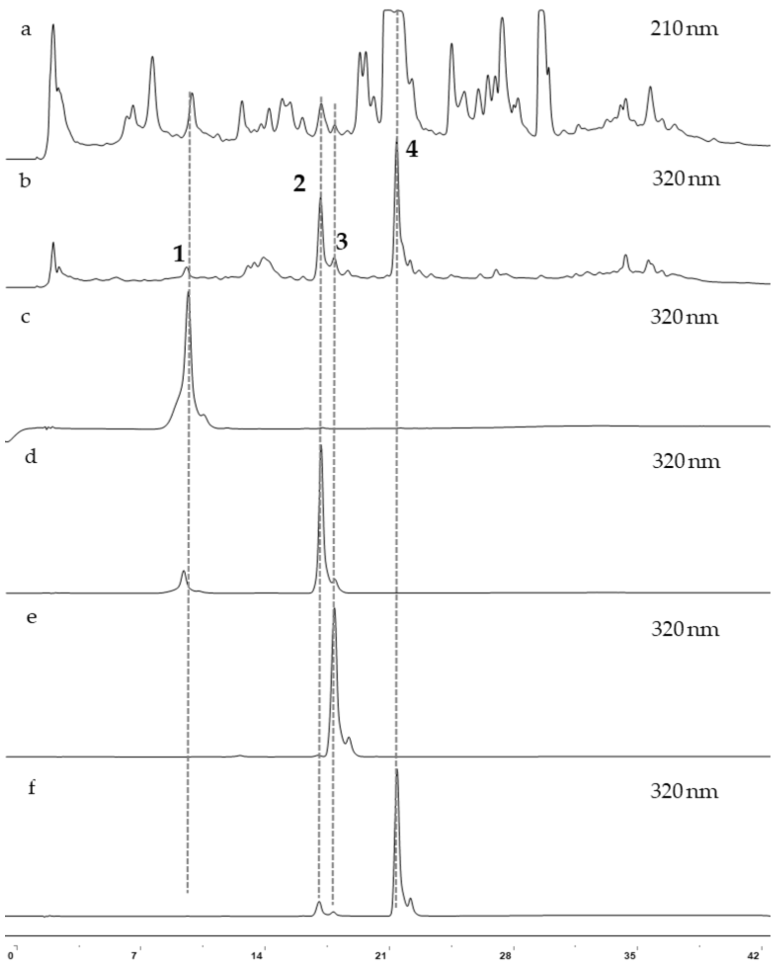

Figure S1. HPLC traces of the crude extract and purified pyripyropenes. **(a**) the ethanol extract detected at 210 nm; (**b**) the ethanol extract detected at 320 nm; (**c**–**d**) compounds 1–4 detected at 320 nm.

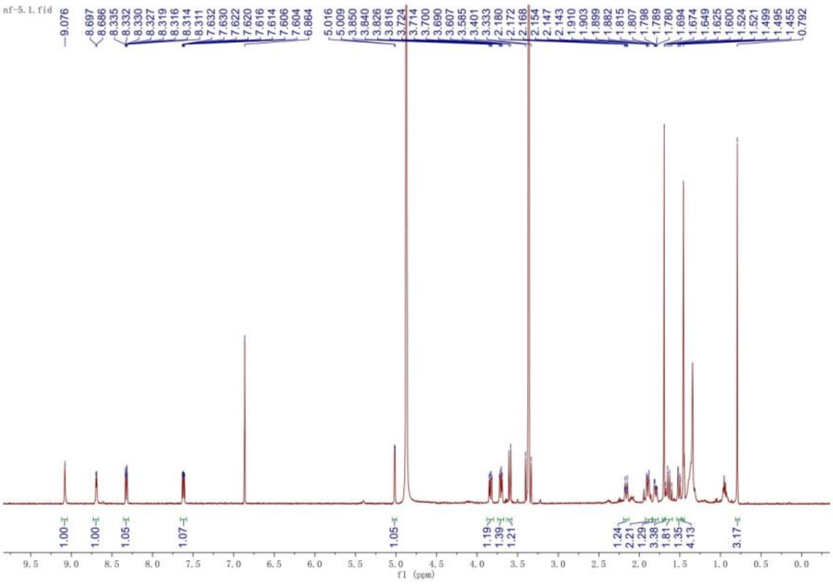

Figure S2. ^{1}H NMR spectrum of 1 in MeOD.

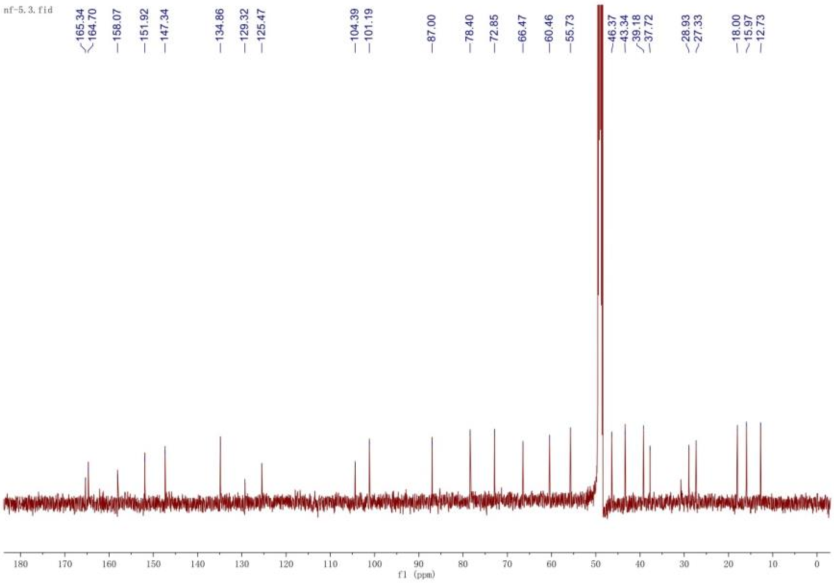

Figure S3. ^{13}**C** NMR spectrum of **1** in MeOD.

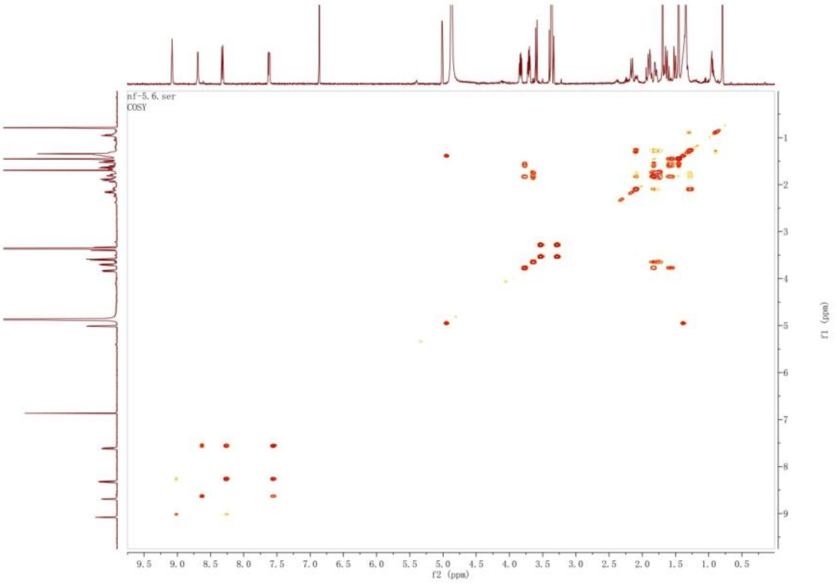

Figure S4. ^{1}H-^{1}H COSY spectrum of **1** in MeOD.

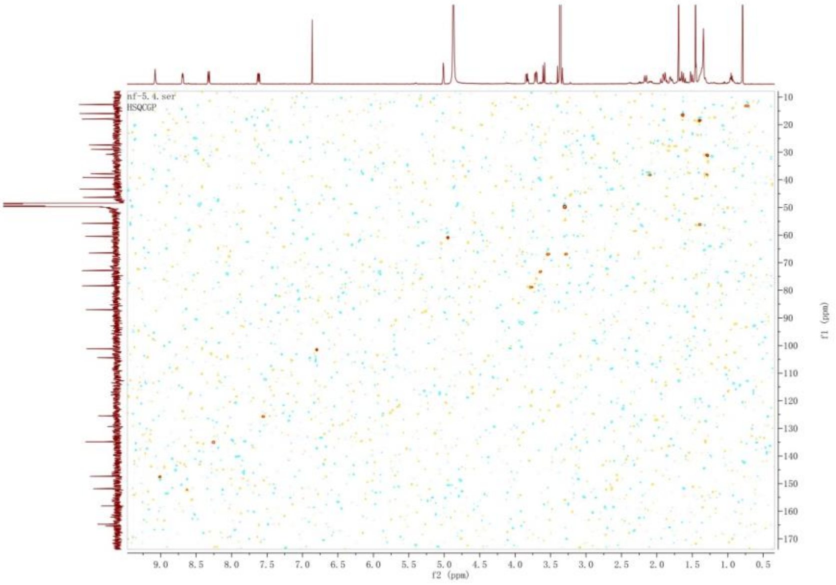

Figure S5. HSQC spectrum of **1** in MeOD.

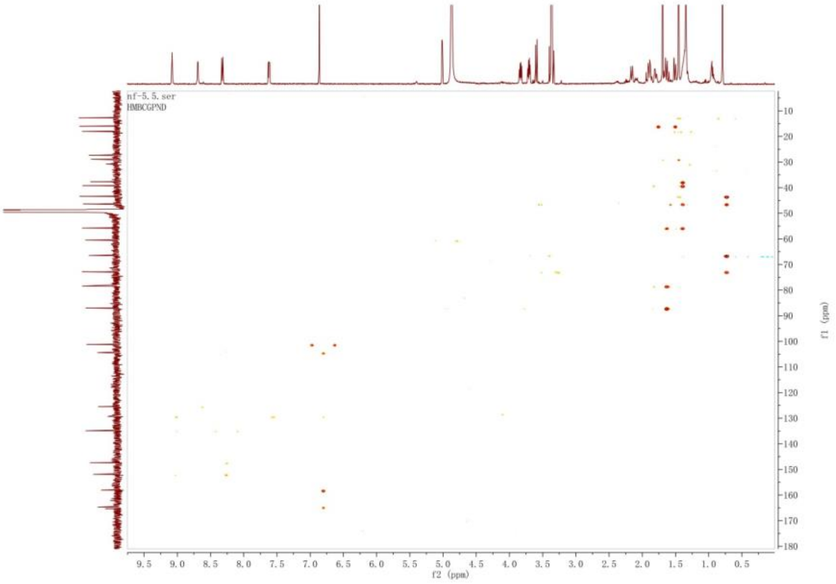

Figure S6. HMBC spectrum of **1** in MeOD.

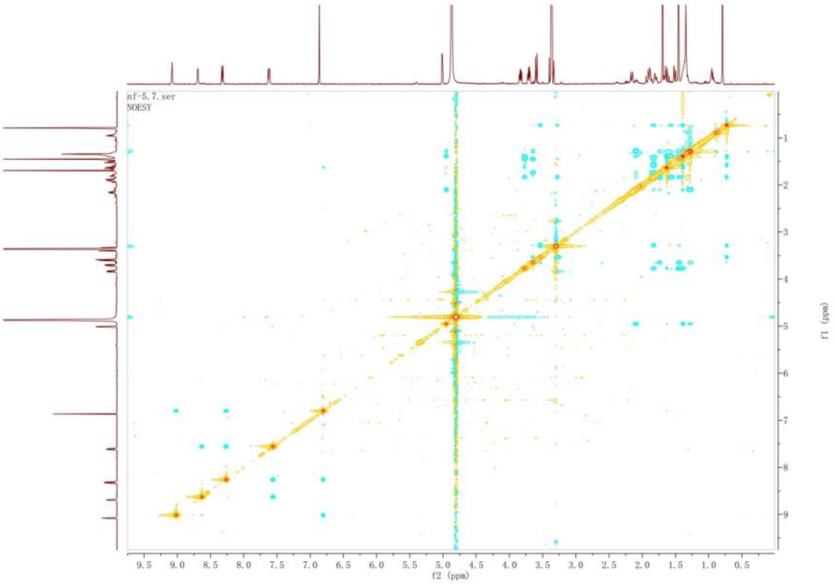

Figure S7. NOESY spectrum of **1** in MeOD.

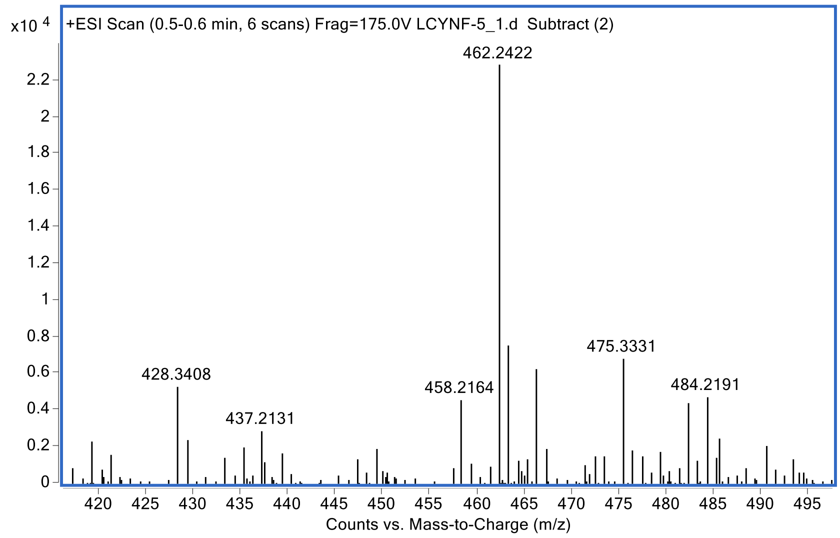

Formula (M)	Ion Formula	m/z	Calc m/z	Diff (ppm)	DBE
C25 H31 N O7	C25 H32 N O7	458.2164	458.2173	2.03	11

Figure S8. HR-ESI-MS spectrum of **1**.

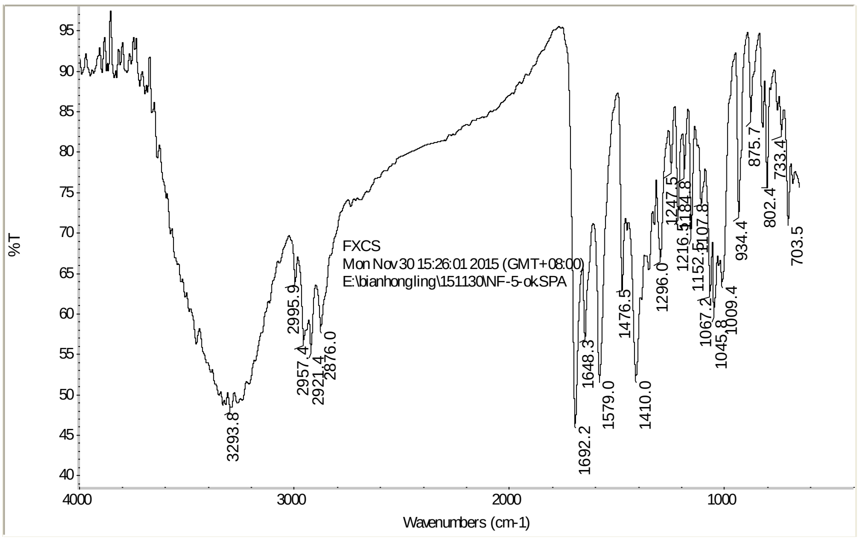

Figure S9. IR spectrum of **1**.

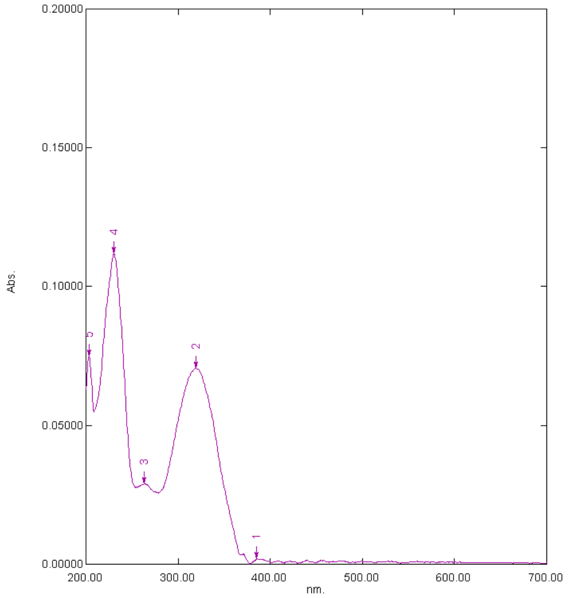

Figure S10. UV spectrum of **1** in MeOD.

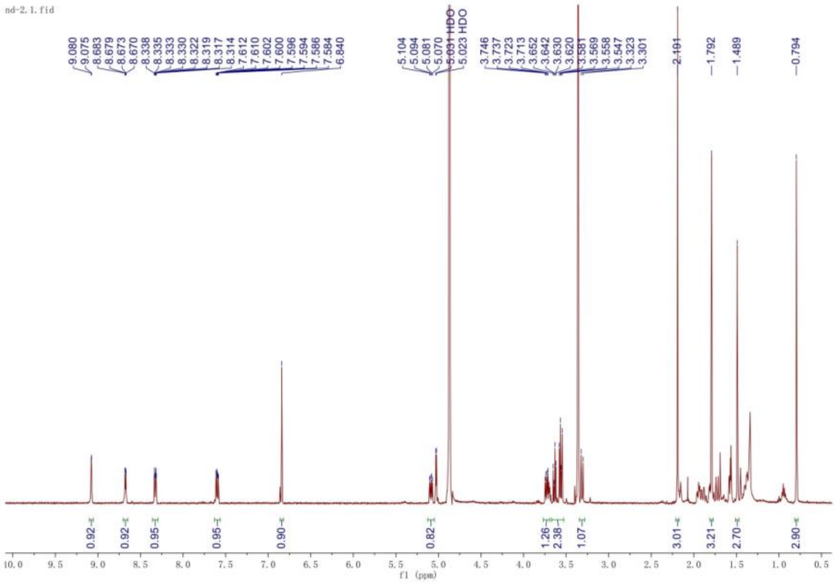

Figure S11. ^{1}H NMR spectrum of **2** in MeOD.

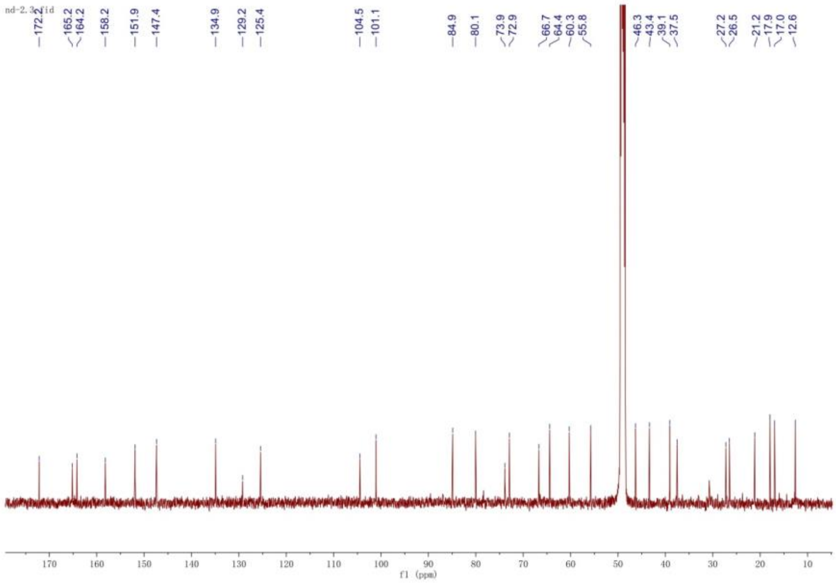

Figure S12. ^{13}C NMR spectrum of **2** in MeOD.

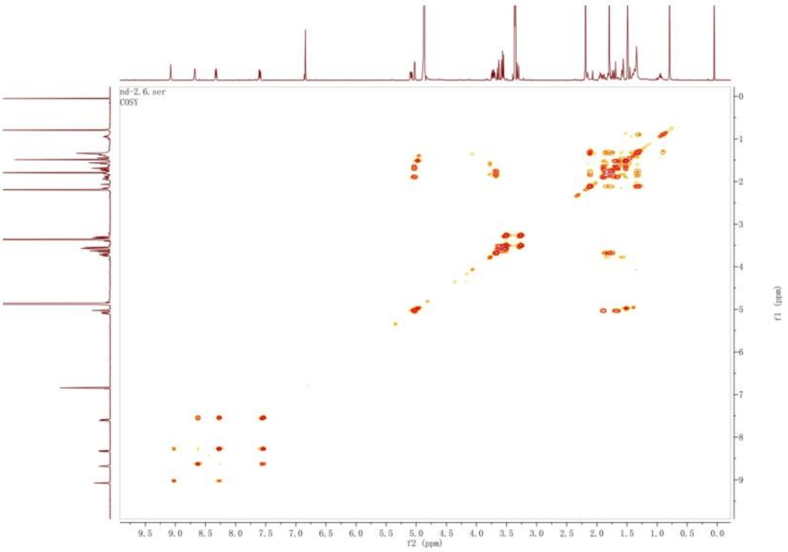

Figure S13. ^{1}H-^{1}H COSY spectrum of **2** in MeOD.

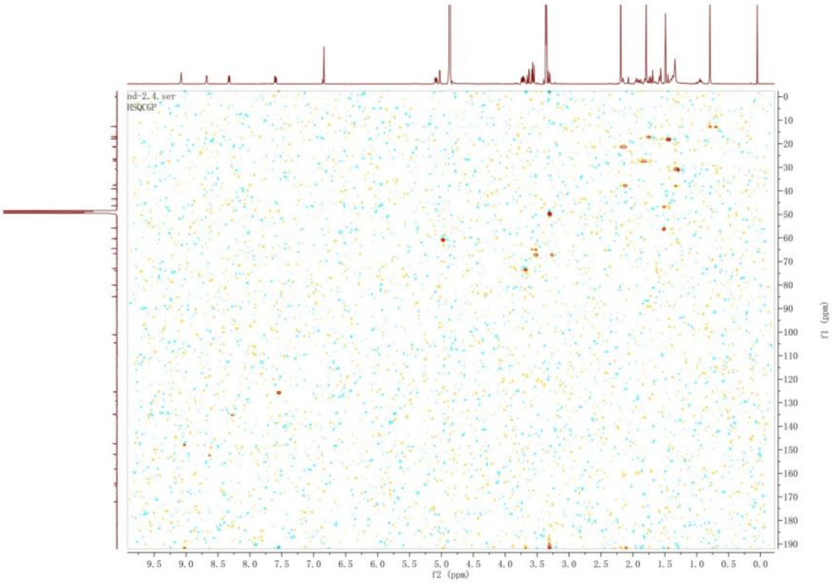

Figure S14. HSQC spectrum of **2** in MeOD.

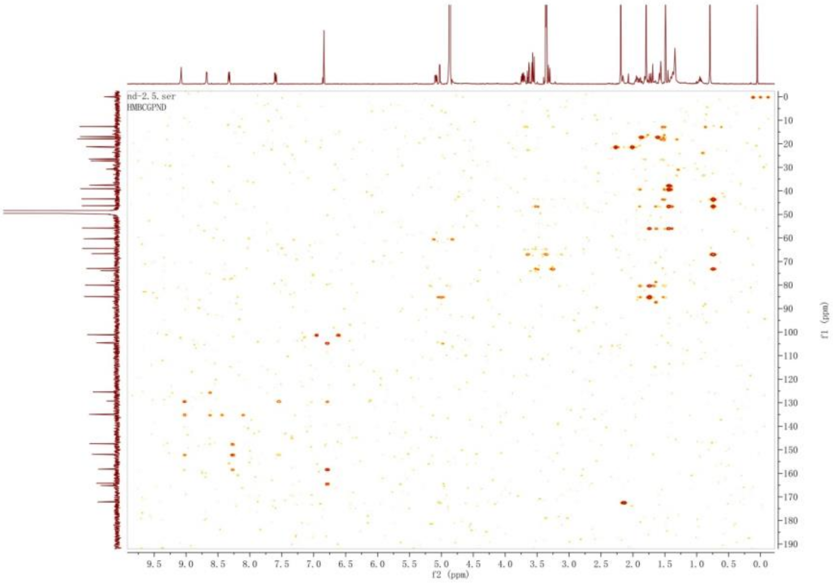

Figure S15. HMBC spectrum of **2** in MeOD.

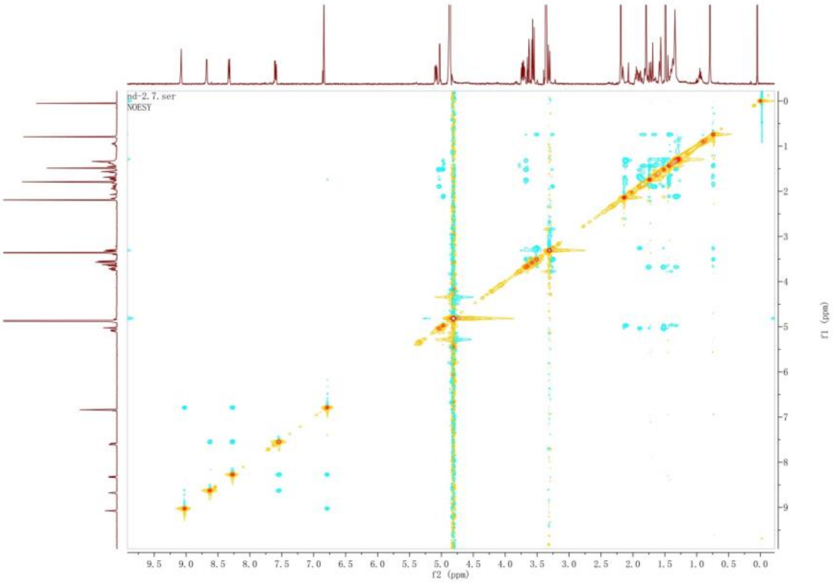

Figure S16. NOESY spectrum of **2** in MeOD

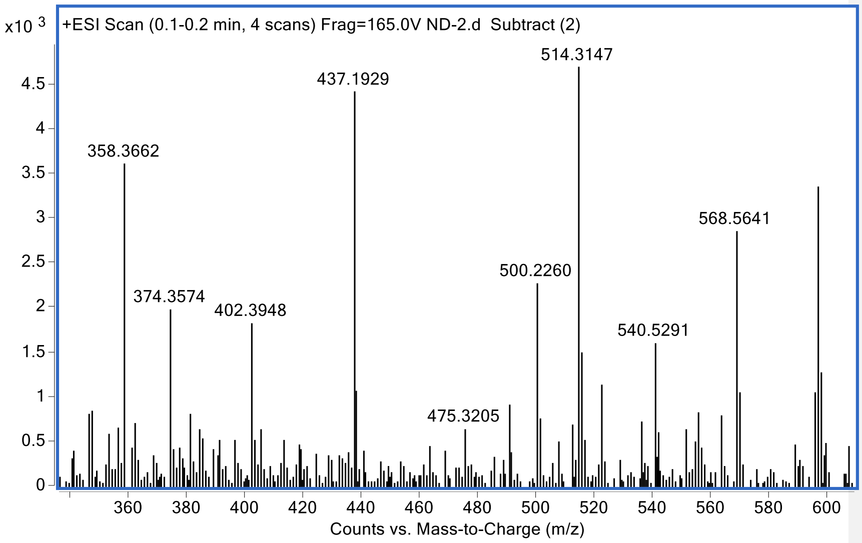

Formula (M)	Ion Formula	m/z	Calc m/z	Diff (ppm)	DBE
C27 H33 N O8	C27 H34 N O8	500.226	500.2279	3.79	12

Figure S17. HR-ESI-MS spectrum of **2**.

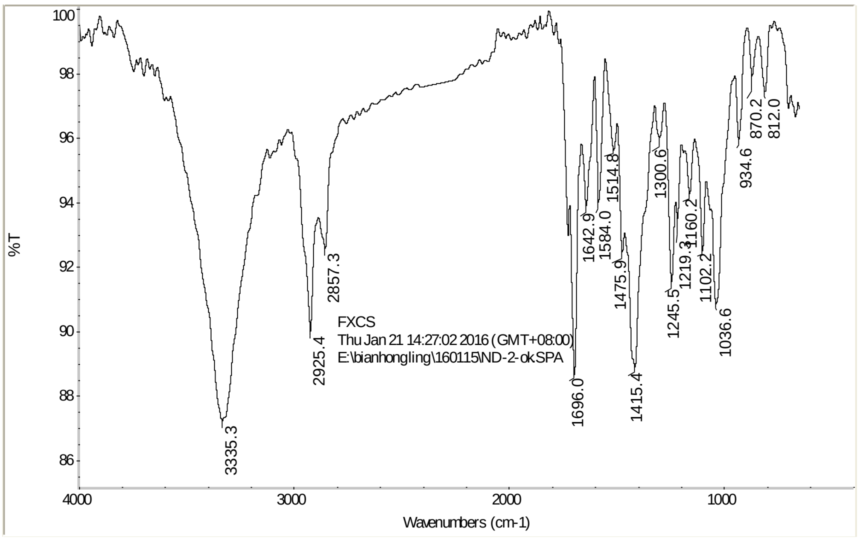

Figure S18. IR spectrum of **2**.

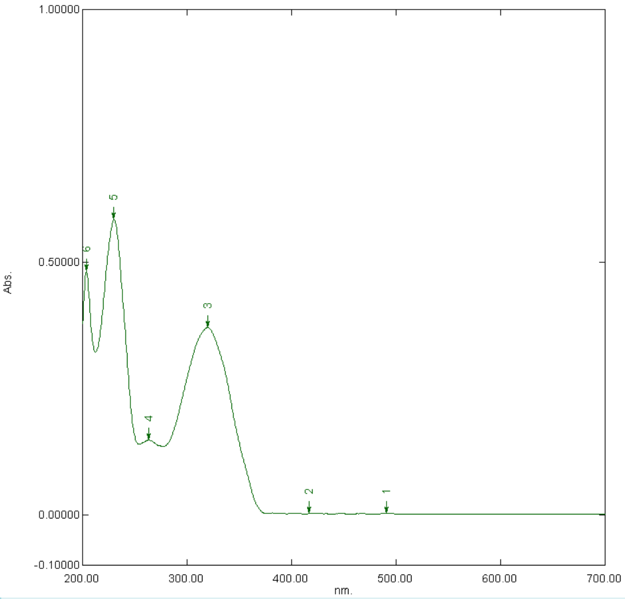

Figure S19. UV spectrum of **2**.

Commented [RF1]: Be sure to use en dashes rather than hyphens for negative numbers.
